# Ordinal Random Processes

**DOI:** 10.3390/e27060610

**Published:** 2025-06-07

**Authors:** Christoph Bandt

**Affiliations:** Institute of Mathematics, University of Greifswald, 17487 Greifswald, Germany; bandt@uni-greifswald.de

**Keywords:** ordinal pattern, stochastic process, time series, permutation entropy, 37A35, 62M10, 60G07

## Abstract

Ordinal patterns have proven to be a valuable tool in many fields. Here, we address the need for theoretical models. A paradigmatic example shows that a model for frequencies of ordinal patterns can be determined without any numerical values. We specify the important concept of stationary order and the fundamental problems to be solved in order to establish a genuine statistical methodology for ordinal time series.

## 1. Introduction

### 1.1. The Main Question

Time series analysis has two parts. First, there are methods for describing data series, like means, correlations, and Fourier spectra. Second, there are models, like Brownian motion or ARMA models, which describe mechanisms that could generate similar sequences of data points. Most models postulate that the next state $X_{t+1}$ is a function $f(X_{t},X_{t-1},\ldots )$ of previous states, with some random influence. A fundamental example is a simple random walk, which states that $X_{t+1}$ is either $X_{t}+1$ or $X_{t}-1,$ depending on the result of a coin toss.

In recent years, the study of ordinal patterns in time series has proven successful for many practical applications. However, there is a lack of appropriate models. Established numerical models provide little information on the ordinal structure. This paper proposes directly constructing proper ordinal models. We raise the following question:

*Can we define mechanisms that explain the order relations between data points in a time series without using any numerical values?*

We shall give an affirmative answer, providing an example of a coin-tossing order. It is similar to a simple random walk. Coin throws are used to define the order relation between all data points while the values, $X_{t}$, remain undefined. This is a completely new type of model. Due to its simplicity, it can be easily understood. To model real-world data, the example is too simple. It must be modified and extended. Toward this end, we provide a mathematical framework in the last section.

### 1.2. Contents of the Paper

In this introductory section, we briefly introduce the basic concepts. For more details and background, we refer the reader to [1] [Section II] and [2,3,4,5,6,7]. One new point involves the visualization of the distribution of ordinal patterns by refining ordinal histograms in Section 1.5. The main topic of this paper is explained in Section 1.7. Order-related properties of processes, particularly order self-similarity, are introduced in Section 1.8.

Section 2 deals with a paradigmatic example of an ordinal process that has no numerical values. The construction is algorithmic and intuitive, and the pattern probabilities for length 3 match those of Brownian motion. Unfortunately, the example is not self-similar and is not relevant to applications. We conjecture that there is a self-similar modification.

Section 3 provides a more technical outline of the possible theory of ordinal processes. We construct stationary ordinal processes without using numerical values. Order self-similarity remains an open problem.

### 1.3. Permutations as Patterns in Time Series

A permutation π of length *m* is a one-to-one mapping from the set {1,2,…,m} onto itself. We write $\pi_{k}$ for π(k) and $\pi =\pi_{1}\pi_{2}\ldots \pi_{m}.$ Thus, π=231 means π(1)=2,π(2)=3,π(3)=1. However, we do not need mapping properties of permutations, like composition. We just consider the graph of π as a geometric pattern (an ordinal pattern).

With $x=x_{1},x_{2},\ldots ,x_{T}$ denote a time series of length T. This can also be viewed as a mapping that assigns to the time points t=1,…,T the values $x_{t}=x\left(t\right).$ For *m* time points $t_{1}<t_{2}<\ldots <t_{m}$ between 1 and *T*, we consider the pattern of corresponding values. We define

(1) $$\left(x_{t_{1}},x_{t_{2}},\ldots ,x_{t_{m}}\right)\;\mathrm{shows}\;\mathrm{pattern}\;\pi \;\mathrm{when}\;x_{t_{i}}<x_{t_{j}}\;\mathrm{if}\;\mathrm{and}\;\mathrm{only}\;\mathrm{if}\;\pi_{i}<\pi_{j}\;.$$

In other words, the correspondence $x_{t_{j}}\to \pi_{j},j=1,\ldots ,m$ is strictly monotonous. This is very intuitive. The pattern π can be determined from the time series by calculating ranks; $\pi_{k}$ is the number of time points $t_{j}$ for which $x_{j}\leq x_{k}.$

### 1.4. Pattern Frequencies in Time Series

Instead of arbitrary $t_{j},$ we study equally spaced time points t<t+d<t+2d<…<t+(m−1)d. We call *m* the length and *d* the delay or lag of the pattern. We always take m≤6 and mainly consider m=3 and m=4. However, we vary *d* as much as possible.

Ordinal pattern analysis is a statistical method. We fix a length, *m*, and determine relative frequencies of all patterns, π, of length m, for various delays, d. We divide the number of occurrences of π by the number of time points where π could occur.

(2) $$p_{\pi }\left(d\right)=\frac{1}{T-(m-1)d}\;\#\left\{t\;|\;1\leq t\leq T-\left(m-1\right)d,\;\left(x_{t},x_{t+d},\ldots ,x_{t+(m-1)d}\right)\;\mathrm{shows}\;\mathrm{pattern}\;\pi \right\}\;.$$

These frequencies are the basic parameters of ordinal time series analysis. They can be combined to calculate permutation entropy, persistence, and other important parameters [1,2,3,4,5,6,7].

### 1.5. Visualization of Pattern Frequencies by Ordinal Histograms

**Figure 1:**
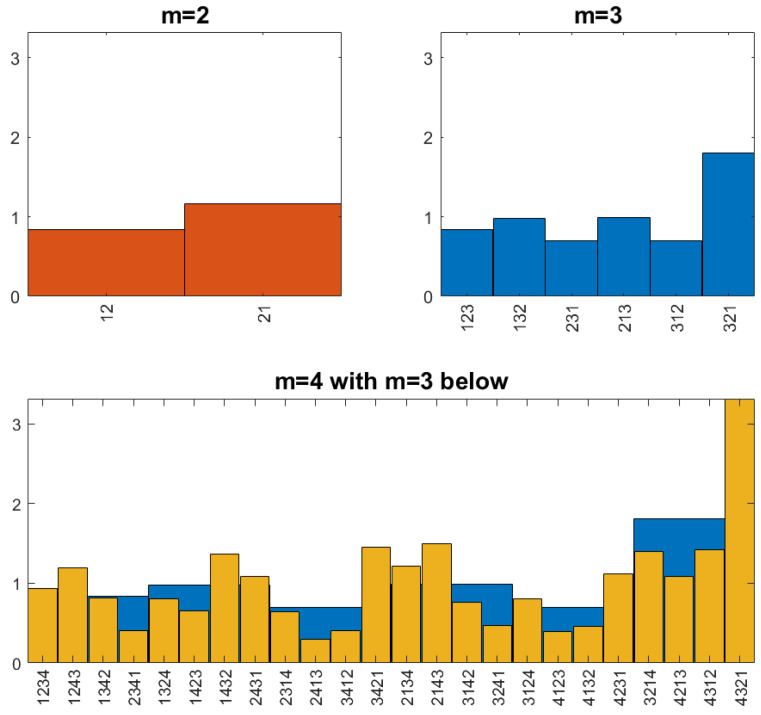
Histograms of pattern frequencies of a simulated time series for d=1 and m=2 (red), 3 (blue), and 4 (brown). Each pattern π of length *m* is represented by a subinterval of [0,1] with length 1/m!. The corresponding bar has area $p_{\pi }\left(d\right),$ so the height is $m!p_{\pi }\left(d\right).$ The permutations are arranged in hierarchical order so that each histogram is a refinement of the previous one. For details, see Section 3.4.

This paper is not about parameters like entropy. It studies models for all pattern frequencies together. For this reason, we introduce a graphical method that summarizes the pattern frequencies for a fixed *d* in a kind of density function. The permutations π are assigned subintervals $I_{\pi }$ of [0,1] as follows: $I_{12}=\left[0,\frac{1}{2}\right],\;I_{21}=\left[\frac{1}{2},1\right].$ We draw bars over $I_{\pi }$, each with area $p_{\pi }\left(d\right).$ For m=3, we note that $p_{12}=p_{123}+p_{132}+p_{231}$ (neglecting a possible tiny error due to the last value for $p_{12}\left(d\right)$ in (2)). We choose $I_{123}=\left[0,\frac{1}{6}\right]$, $I_{132}=\left[\frac{1}{6},\frac{1}{3}\right],\;I_{231}=\left[\frac{1}{3},\frac{1}{2}\right]$ so that their union is $I_{12}.$ Then we draw bars with area $p_{\pi }\left(d\right).$ The same is done for π=213,312,321. In this way, the histogram for m=3 is a refinement of the histogram for m=2, in the sense that the area above $I_{12}=\left[0,\frac{1}{2}\right]$ remains the same. In theory, successive refinements can be obtained for m=4,5,… but in practice we rarely have reliable estimates for m>4. This arrangement of permutations is called the hierarchical order. Details will be given in Section 3.4. As Figure 1 shows—for a simulated time series—all pattern frequencies of a time series for a fixed *d* can be subsumed in this way by a sequence of refining histograms, and in the limit m→∞ by a density function.

The process chosen for Figure 1 is an AR(1) model with exponential noise: $X_{t+1}=\frac{1}{2}X_{t}-\log U_{t}$, where the $U_{t}$ are independent uniformly distributed random numbers in [0,1]. Due to the asymmetry of the noise distribution, $p_{21}\left(1\right)\approx 0.58>\frac{1}{2}.$ Successive decreasing steps are frequent: $p_{321}\left(1\right)\approx 0.3$ and $p_{4321}\left(1\right)\approx 0.13.$ We cannot explain why 2143 is the permutation with the second-largest frequency. However, an AR(1) model with the coefficient $\frac{1}{2}$ has a short memory. For d=3, the patterns already have almost uniform frequencies. The models considered below are more uniform for m=2 and 3, but they also provide interesting behavior for large d.

**Figure 3:**
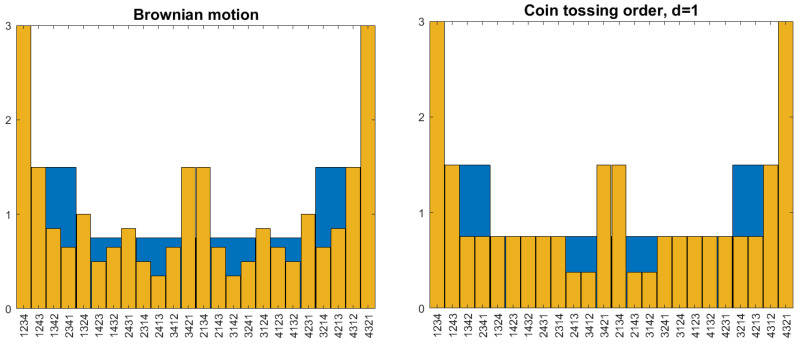
Probabilities of patterns of length 4 (brown) on top of those of length 3 (blue) for the Brownian motion (**left**) and coin-tossing order with d=1 (**right**). For length 3, the probabilities coincide.

The objective of this paper is to find typical ordinal histograms from a theoretical viewpoint. An important objective is to find models where the histograms agree for all d. Two such models are known [1] [Section II.C], that is, white noise, where the histogram is a constant function for all m, and Brownian motion; see Figure 3. We are looking for other models.

### 1.6. Stationary and Order Stationary Processes

When modeling time series, we suppose that the given time series is an instance of a larger ensemble, called a stochastic process. The process represents the mechanism that generates the given time series and many other ones. We want to know the laws of this process. Formally, a stochastic process, *X*, is a sequence, $X=X_{1},X_{2},\ldots$, of real random variables on a probability space (Ω,P). The time series, *x*, is a random choice from the ensemble X.

To find the laws of *X* from a single time series, we must assume that the distributions and dependencies of $X_{k}$ do not change in time. Usually, *X* is required to be stationary. That is, the *m*-dimensional distribution of $\left(X_{t},X_{t+1},\ldots ,X_{t+m}\right)$ does not depend on t, for all m. This assumption is very strong. When we study autocorrelation, we assume weak stationarity. That is, the mean $M(X_{t})$ and the covariance $Cov(X_{t},X_{t+k})$ do not depend on *t* [8]. When we consider ordinal patterns, we assume a similar, very weak stationarity assumption. It is required throughout this paper.

**Order stationarity.** For a given pattern π with length *m* and delay d, let

(3) $$P_{\pi }\left(d\right)=P\left\{\left(X_{t},X_{t+d},\ldots ,X_{t+(m-1)d}\right)\;\mathrm{shows}\;\mathrm{pattern}\;\pi \right\}$$

denote the probability that the process, *X*, shows pattern π just after time t. We say that the process, *X*, is *order stationary for patterns of length m* if $P_{\pi }\left(d\right)$ is the same value for all t. This should hold for all patterns, π, of length *m* and all delays, d. For a time series of length T, we consider only $d=1,\ldots ,d_{\max}$, where $d_{\max}$ must be smaller than T/m.

Brownian motion (where $X_{0}=0$ and $X_{t+1}-X_{t}$ are independent increments with standard normal distribution for t=0,1,…) is an order stationary process that is not even weakly stationary. It is a basic model for the financial time series. Note that the process, *X*, and the pattern probabilities, $P_{\pi }\left(d\right)$, are theoretical objects. The observed frequency $p_{\pi }\left(d\right)$ serves as an estimate of $P_{\pi }\left(d\right),$ and from a theoretical perspective, the estimator Formula (2) has nice properties [9,10,11,12,13]. The practical success of ordinal patterns indicates that the assumption of order-stationarity is at least approximately fulfilled in applications.

### 1.7. The Topic of This Paper

There are plenty of well-studied stochastic processes, including Gaussian processes, random walks, ARMA and GARCH processes, processes with long-range dependence [11,14], etc. Most of them are either stationary or have stationary (multi-dimensional) increments, and are, therefore, order stationary. However, they are defined by arithmetical operations. This makes the rigorous study of ordinal pattern frequencies difficult, although various interesting theorems could be derived [9,10,11,15,16]. Even for the Brownian motion, exact frequencies of patterns can be determined only for m≤4 [17]. Some of them are rational, and some are irrational. Frequencies for length 5 lead to integrals that can only be numerically calculated. Overall, the impression is that ordinal patterns do not fit into the established theory of stochastic processes.

The only proper theoretical model for pattern frequencies is white noise, which is a process consisting of independent and identically distributed random variables, $X_{i}.$ For each m, all permutations have the same probability, $p_{\pi }=1/m!$, for any delay, d. This model was repeatedly used as a null hypothesis for testing the serial dependence of time series [1,12,13,18,19,20,21]. A greater variety of models is needed.

In this paper, we attempt to define ordinal random processes in a combinatorial way, directly by their ordinal pattern frequencies. We do not require normal distribution, we require no arithmetic, and we do not even need numerical values to compare objects $X_{i}$ and $X_{j}.$ An algorithmic example in the next section will show that this is possible. In Section 3, we outline some technical concepts and basic statements for a theory of ordinal processes. This could be the first step toward finding more applicable ordinal models. Note that the possible equality of values, an important problem in practice [21], plays no role in this paper; our models will only allow $X_{s}<X_{t}$ or $X_{s}>X_{t}$ but not $X_{s}=X_{t}.$

### 1.8. Symmetry and Independence Properties of Ordinal Processes

Of course, it is not enough to just postulate pattern frequencies. Our models should also have nice properties. Here, we list the ordinal version of some of the most common process properties. We start with symmetry in time and space [9].

A process, *X*, is reversible if the distributions of $\left(X_{1},\ldots ,X_{m}\right)$ and $\left(X_{m},\ldots ,X_{1}\right)$ agree for every m. In the ordinal version, it is reversible if the permutations $\pi =\pi_{1}\pi_{2}\ldots \pi_{m}$ and $\pi_{m}\pi_{m-1}\ldots \pi_{1}$ have the same pattern probability for every π. The process, *X*, is invariant under the reversal of values if the distributions of $\left(X_{1},\ldots ,X_{m}\right)$ and $\left(-X_{1},\ldots -X_{m}\right)$ agree for every m. The ordinal version states that the permutations $\pi =\pi_{1}\pi_{2}\ldots \pi_{m}$ and $\left(m+1-\pi_{1}\right)\left(m+1-\pi_{2}\right)\ldots \left(m+1-\pi_{m}\right)$ have the same pattern probability for every π.

In both cases, the ordinal versions of symmetry are much weaker and easier to check. These symmetry properties are often met in applications. As a consequence, the distribution of ordinal patterns of length 3 often has only one degree of freedom, since $p_{123}=p_{321},$ and the probabilities of the other four permutations agree. All Gaussian processes are symmetric in space and time [9,11,17].

A process, *X*, is said to be Markov if for any *t* and fixed $X_{t},$ the distributions of $\left(X_{1},\ldots ,X_{t-1}\right)$ and $\left(X_{t+1},X_{t+2},\ldots \right)$ are independent. In the ordinal version, this means that the appearances of patterns $\pi ,\pi^{\prime }$ at times $t_{1}<\ldots <t_{m}\leq t$ and $t\leq t_{1}^{\prime }<\ldots <t_{m}^{\prime },$ respectively, are independent. Again, the ordinal property is weaker and easier to check. The Markov property appears in theory more than in applications.

**Order self-similarity.** The most important symmetry property in this paper is order self-similarity [22] [Section 6]. A random process in continuous time, $Y_{t},t>0$, is said to be *self-similar* if there is an exponent, H>0, such that $Y_{rt}=r^{H}Y_{t}$ in distribution, for each positive number, r. Such a process cannot be stationary, it must look similar to Brownian motion, and it has a very special mathematical structure [23]. The ordinal concept for discrete time is much weaker, more general, and very natural. It does not depend on a parameter, and it can be statistically checked for real-world data.

A process, *X*, is said to be *order self-similar for patterns π of length m* if

(4) $$P_{\pi }\left(d\right)\;\mathrm{is}\;\mathrm{the}\;\mathrm{same}\;\mathrm{value}\;\mathrm{for}\;\mathrm{all}\;d.$$

This should hold for all patterns, π, of length *m* and all delays, d.

For a time series of length T, we consider $d=1,\ldots ,d_{\max}$, where $d_{\max}$ is much smaller than T. With such a restriction, order self-similarity was statistically verified in financial time series [22] and EEG measurements [1]. For self-similar Gaussian processes with stationary increments, Sinn and Keller [9] estimated the Hurst exponent, *H*, from pattern frequencies. For real-world data, however, it is not at all clear to what extent the strong self-similarity is fulfilled and the exponent *H* is meaningful. Thus, for practical purposes, order self-similarity seems to be the right concept to start with. The present paper is motivated by the desire for order self-similar models.

## 2. The Coin-Tossing Order—An Ordinal Process Without Values

### 2.1. Ranking Fighters by Their Strength

Ordinal patterns were derived from numerical values of a time series. Now, we go the opposite way, similar to the world of tennis or chess, where players are first compared pairwise and then assigned a rank or ELO score. Suppose there are four players, A,B,C,D, in a tennis club. We design an algorithm to order them by strength. First, *B* plays against A. Let us assume *B* wins. Then we write A<B. Now the next player, *C*, plays against the previous player, B. If *C* wins, then A<B<C by transitivity of order. If *C* loses, however, *C* must still play against A. Depending on the result, either A<C<B or C<A<B. Now, the last player, *D*, comes and plays first against the previous player, C, and then against *B* and/or A, if the comparison is not yet determined by previous games and transitivity.

This procedure is turned into a mathematical model. We want to construct a random order between objects $X_{1},X_{2},\ldots$, which are not numbers. We follow the algorithm for tennis players and replace each match with the toss of a fair coin. As a result, we obtain a stochastic process like white noise or Brownian motion on the ordinal level only. It is possible to determine pattern probabilities much better than for the Brownian motion.

### 2.2. Definition of the Coin-Tossing Order

Repeated coin tossing is a standard way to represent randomness. Take a simple random walk, for example, and write $X_{n}$ for our position at time n=0,1,2,… then $X_{0}=0,$ and either $X_{n+1}=X_{n}+1$ or $X_{n+1}=X_{n}-1$, depending on the result of a coin throw at time n. In the present case, $X_{1},X_{2},X_{3},\ldots$ will denote objects, not necessarily numbers. We define a random order between the $X_{i}.$ We throw a fair coin, $c_{ji}$, to decide whether $X_{i}<X_{j}$ or $X_{i}>X_{j},$ for any pair of integers, i<j. Let us write 1 for ‘head’ and 0 for ‘tail’. Then our basic probability space Ω is the space of all 0-1-sequences, where each coordinate is 0 or 1 with probability $\frac{1}{2},$ independently of all other coordinates. Formally,

$$\Omega =\{\left(c_{21},c_{32},c_{31},c_{43},c_{42},\ldots \right)\;|\;c_{ji}\in \left\{0,1\right\}for\;j>i\geq 1\}$$

where $c_{ji}=0$ means $X_{i}<X_{j},$ and $c_{ji}=1$ means $X_{i}>X_{j},$ for 1≤i<j. The important point is that $c_{ji}$ is disregarded when the order of $X_{i}$ and $X_{j}$ is already fixed by previous comparisons and transitivity.

The first coin $c_{21}$ decides the ordering of $X_{1}$ and $X_{2}.$ Now suppose $X_{1},\ldots ,X_{j-1}$ are already ordered. Then, $X_{j}$ is compared to $X_{j-1},X_{j-2},\ldots ,X_{1}$ by considering the random numbers $c_{ji}.$ However, when the comparison is fixed by transitivity from the already defined ordering, then $c_{ji}$ is disregarded—that coin need not be thrown.

**Figure 2:**
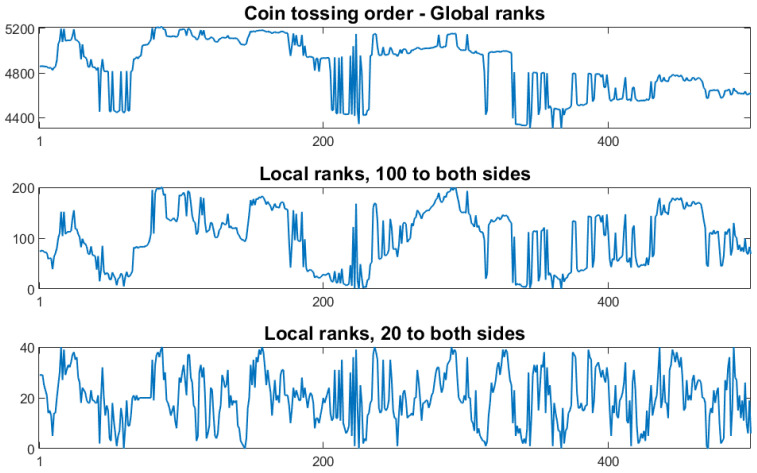
Global and local rank numbers obtained from coin tossing.

The resulting random order is called the *coin-tossing order.* It can be easily simulated. Figure 2 shows the rank numbers of 500 consecutive objects, $X_{j}$, in the middle of a simulated series of length T = 10,000. The global rank numbers have strange discontinuities. Local rank numbers, obtained by comparing with the following 20 objects on the left and right sides, show a more familiar picture.

**Problem.** Is there a stochastic process with stationary increments that generates the coin-tossing order?

### 2.3. Basic Properties of the Coin-Tossing Order

Despite the erratic view of trajectories in Figure 2, it turns out that the coin-tossing order has the same ordinal pattern frequencies for length 3 as Brownian motion, for arbitrary d. In particular, it is order self-similar for patterns of length 3.

**Theorem** **1** (Basic properties of the coin-tossing order).

*(i)*  *The coin-tossing order is stationary and has the ordinal Markov property.*
*(ii)*  *For any permutation, π, of length m, the pattern probability is $P_{\pi }\left(1\right)=2^{-u}$ with*
  (5) $$u=u\left(\pi \right)=\#\;\{\left(i,j\right)|\;1\leq i<j\leq m,\;if\;i<k<j\;then\;\pi_{k}\;is\;not\;between\;\pi_{i}\;and\;\pi_{j}\}\;.$$
*(iii)*  *The pattern probabilities, $P_{\pi }\left(d\right)$, are invariant under time reversal and the reversal of values.*
*(iv)*  *For d=1,2,…. we have $P_{12}\left(d\right)=\frac{1}{2},\;P_{123}\left(d\right)=P_{321}\left(d\right)=\frac{1}{4},$ and $P_{\pi }\left(d\right)=\frac{1}{8}$ for the other permutations of length 3.*

**Proof.**  (i): The order of $\left(X_{t},X_{t+1},\ldots ,X_{t+m-1}\right)$ depends on the random numbers $c_{ji},t+m-1\geq j>i\geq t,$ in the same way that the order of $\left(X_{1},X_{2},\ldots ,X_{m}\right)$ depends on the $c_{ji},m\geq j>i\geq 1.$ Since both collections of random numbers have the same distribution, the pattern probabilities do not depend on t, and the defined random order is stationary. Moreover, the comparisons of $X_{s}$ with s≥t depend on random numbers $c_{ji}$ with j>t while the comparisons of $X_{s}$ with s≤t depend on $c_{ji}$ with j≤t. Since the different $c_{ji}$ are independent, this implies the ordinal Markov property.

(ii): u(π) is the number of coin flips needed to determine π. Given a permutation π, we determine the $c_{ji}$ variables, which are needed to define the occurrence of π in $(X_{1},X_{2},\ldots ,X_{m}).$ Of course, m≥j>i≥1, and *j* run in increasing order. For the fixed j, the number $c_{j,j-1}$ is always used, and the other *i* variables are considered in decreasing order. Now, consider *k* with i<k<j. If $\pi_{i}<\pi_{k}<\pi_{j},$ this is determined by the random numbers $c_{ki}$ and $c_{jk}$, which were drawn before $c_{ji}.$ In that case, $c_{ji}$ is disregarded since $\pi_{i}<\pi_{j}$ follows from the transitivity of the order. A similar argument applies when $\pi_{i}>\pi_{k}>\pi_{j}.$ However, if there is no $\pi_{k}$ between $\pi_{i}$ and $\pi_{j},$ then $c_{ji}$ is needed to determine π. We shall call u(π) the energy of π.

(iii): First, we consider d=1. Given π, we have to show that the time-reversed and spatially reversed permutations have the same energy u(π). For spatial reversal, this directly follows from the definition (5) with ‘between’. For time reversal, we show that $P_{\pi }\left(1\right)$ can also be determined backwards by considering $c_{ji}$ with decreasing i=m−1,m−2,…,1, and for fixed i, with increasing j=i+1,…,m. The point is that when we compare places, i<j, we have already compared both *j* and *i* with all *k* between. So, $c_{ij}$ is needed only if no $\pi_{k}$ is between $\pi_{i}$ and $\pi_{k}.$ Otherwise, the order between $\pi_{i}$ and $\pi_{j}$ is already fixed, and $c_{ij}$ is disregarded. This proves reversibility for d=1.

Now consider d>1 and π of length *m*, which appears as a pattern of $(X_{1},X_{1+d},\ldots ,X_{1+(m-1)d}).$ The probability of this event is $P_{\pi }\left(d\right)$, which may be different from $P_{\pi }\left(1\right).$ We have to calculate $P_{\pi }\left(d\right)$ from all ‘atom permutations’ of length M=1+(m−1)d for $\left(X_{1},X_{2},\ldots ,X_{1+(m-1)d}\right)$ for which the order among the special *m* places agrees with the order of π. This would be a lot of work. However, since the reversed permutation of π is composed of the reversed ‘atom permutations’, both in space and in time, we shall obtain the same pattern probability.

(iv): The spatial reversal invariance implies $P_{12}\left(d\right)=P_{21}\left(d\right)=\frac{1}{2}.$ Then, $P_{123}\left(d\right)=P_{321}\left(d\right)=\frac{1}{4}$ follows from the Markov property. The equality of the four other pattern frequencies is a consequence of the invariance under time and space reversal.    □

### 2.4. Computer Work and Breakdown of Self-Similarity

In Figure 3, we compare pattern probabilities of length 4 for the Brownian motion, taken from [17], with those of the coin-tossing order. The Brownian motion looks more interesting since coin-tossing allows only the probabilities $2^{-k}.$ Our model is simple, but not recommended for applications. For length 3, the $P_{\pi }\left(d\right)$ of the two processes agree and do not depend on d, according to Theorem 1. The figure shows that we must investigate patterns of length ≥4 if we want to distinguish processes like these two. Moreover, the Brownian motion is perfectly self-similar so that the probabilities, $P_{\pi }\left(d\right)$, of any length do not depend on *d* [23]. Is this also true for the coin-tossing order?

Equation (5) allows for accurate calculation of all pattern probabilities for d=1 and m≤10. Note that such pattern probabilities just for m=5 have never been determined for non-trivial stochastic processes, not even for the Brownian motion or ARMA models of low order [17]. Here is a MATLAB procedure that determines u(x) for a permutation *x*, given as a vector of length m. The vector *w* contains the logical values 0 and 1.

function u=ctoscore(x);

m=length(x); u=m-1; % coin throws for i=1,...,m-1 and j=i+1 are necessary

for i=1:m-2; for j=i+2:m; v=x(i+1:j-1); % look for numbers k between i, j

if x(i)<x(j); w=v>x(i)&v<x(j); else w=v<x(i)&v>x(j); end % x(k) as in (5)

if sum(w)==0; u=u+1; end % if such x(k) do not exist, add a coin throw

end; end;                                      % end of loops for i and j

It takes five minutes to run this procedure over all permutations of length m=10. The minimum number u(x) is m−1=9 and appears only for x=12…m and x=m(m−1)…1. The maximum number u(x)=33 is realized by eight permutations, that is, x=67891012345, and similar patterns. About three-quarters of the 3.6 million patterns fulfill 23≤u(x)≤27.

Now, we can determine $P_{\pi }\left(d\right)$ for all permutations of length 4 and d=3 by adding probabilities $2^{-u(x)}$ of ‘atom permutations’ *x* of length 10, as in the proof of Theorem 1, (iii). For π=1324, we consider all *x* with x(1)<x(7)<x(4)<x(10). We add 10!/4!=151,200 cases. The result must be a rational number with denominator $2^{k}$, where k≤33. For π=1324, we get $P_{\pi }\left(3\right)=3\cdot 41\cdot 59\cdot 10,321/2^{31}\approx 3.49\%.$ To determine $P_{\pi }\left(2\right)$ for patterns of length 4, it suffices to consider m=7.

**Figure 4:**
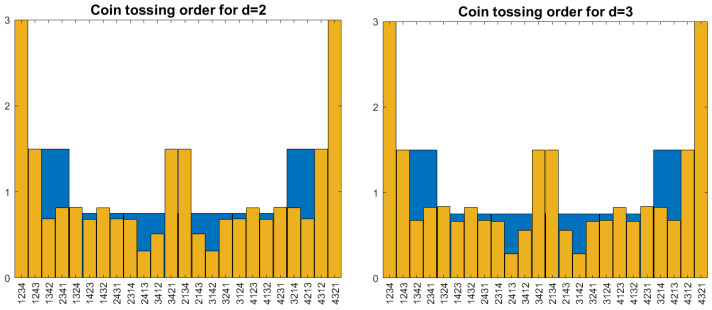
Probabilities of patterns of length 4 for the coin-tossing order with d=2 and d=3. They are clearly different from d=1 while the changes from d=2 to d=3 are small.

The result is shown in Figure 4. Unfortunately, order self-similarity breaks down for patterns of length 4. For π=1324, we have $P_{\pi }\left(1\right)=\frac{1}{32}\approx 3.12\%$ while $P_{\pi }\left(2\right)\approx 3.42\%$ and $P_{\pi }\left(3\right)\approx 3.49\%.$ However, the probabilities for d=2 or 3 can be taken as a new model, which is nearer to self-similarity and looks not as artificial as coin tossing in Figure 3.

**Problem.** Let us define new pattern probabilities $Q_{\pi }\left(1\right)=P_{\pi }\left(d\right)$ for d=2,3,… what are the properties of *Q*? Is there a limit for d→∞ ?

The small difference between d=2 and d=3 in Figure 4 indicates fast convergence. The mere existence of an order self-similar limit would be interesting, but not yet helpful. After all, Brownian motion is already available as an ordinally self-similar process. The merit of the coin-tossing order lies in its algorithmic flavor and its connection to ranking. We look for an ordinally self-similar model with such an intuitive explanation.

Finally, let us consider the interpretation of u(π) as an energy function. Let d=1, and let $S_{m}$ denote the set of all permutations of length m. Let *P* denote the probability measure on $S_{m}$ given by the probabilities, $P_{\pi }=2^{-u(\pi )}$, of the coin-tossing order. The permutation entropy [24] of P, using logarithms with base 2, is just the mean energy with respect to P.

$$H\left(P\right)=-\sum_{\pi }P_{\pi }\log_{2}P_{\pi }=\sum_{\pi }P_{\pi }u\left(\pi \right)=M_{P}\left(u\right)\;.$$

For all other probability measures, *Q* on $S_{m},$ the permutation entropy is smaller than the mean energy; see [25] [Chapter 1]. Thus, *P* is a so-called Gibbs measure on $S_{m}.$

**Problem.** Are there other meaningful energy functions for permutations, perhaps even parametric families of Gibbs measures on $S_{m}$?

## 3. Rudiments of a Theory of Ordinal Processes

### 3.1. Random Stationary Order

Having studied one example, we will now discuss a possible theory of ordinal random processes. As above, $X_{1},X_{2},\ldots$ are not numbers, but only objects that are ordered. We have the discrete-time domain {1,…,T} for time series and N={1,2,…} for models. An order is a relation < on the time domain such that x≮x, and x≠y implies either x<y or y<x, and x<y together with y<z implies x<z. Of course, the order does not apply to the time points t, but to the corresponding objects $x_{t}.$ Then the statement that $x_{t},\ldots ,x_{t+m-1}$ exhibits pattern π has a meaning for every $\pi \in S_{m}$—it is either true or false. When an order on N or {1,…,T} is given, we can determine pattern frequencies, permutation entropy, and so on. In the following, we always have d=1.

We want to construct models of ordinal processes, like the coin-tossing algorithm. For this purpose, we need the concept of random order. To keep things simple, we identify an ordinal process with its law, as follows: *A random order is defined as a probability measure on the set of all orders on the time domain.* For the finite time domain {1,…,T}, a random order is just a probability measure, $P_{T}$, on the set, $S_{T}$, of permutations of length T. For m≤T, $\pi \in S_{m}$, and 1≤t≤T+1−m, the random order allows determining the probability:

(6) $$P_{\pi }^{t}=P\left\{x_{t},\ldots ,x_{t+m-1}\;\mathrm{shows}\;\mathrm{pattern}\;\pi \right\}$$

The random order is called *stationary* if the probabilities $P_{\pi }^{t}$ do not depend on the time point t, for any pattern π of any length m<T. In other words, the numbers $P_{\pi }^{t}$ must be the same for all admissible t. This is exactly the order stationarity that we defined for numerical processes in (3).

### 3.2. The Problem of Finding Good Models

The infinite time domain N is considered in the next section. We use the classical measure theory, which is easy. The real problem appears already for finite T, even for T=20. We have an abundance of probability measures on $S_{T}$ since we can prescribe $P_{\pi }$ for every π. When we require stationarity, we have (T−1)! equations for these T! parameters, as shown below, which still leaves too many choices.

The objective is to select realistic $P_{\pi }.$ Most of the patterns, π, for m=20 will never appear in any real-time series, and we could set $P_{\pi }=0,$ but we do not know for which π. There are three types of properties that we should require for our model.

- Independence: The Markov property or *k*-dependence of patterns (cf. [12]).
- Self-similarity: $P_{\pi }\left(d\right)$ should not depend on d. For processes with short-term memory, like Figure 1, this could be replaced by the requirement that $P_{\pi }\left(d\right)$ converge to the uniform distribution, exponentially with increasing d. There must be a law connecting $P_{\pi }\left(d\right)$ for different d. Otherwise, how can we describe them all?
- Smoothness: There should not be too much zigzag in the time series. Patterns like 3142 should be exceptions. This can perhaps be reached by minimizing certain energy functions.

**Problem.** Does there exist on $S_{100}$ a stationary random order that is Markov and self-similar (for admissible *d* and π) and has parameters different from white noise and Brownian motion; for instance, $P_{12}\neq \frac{1}{2},$ or $\frac{1}{3}<P_{123}+P_{321}<\frac{1}{2}$?

### 3.3. Random Order on N

The infinite time domain N has its merits. Order stationarity, for instance, is very easy to define since every pattern can be shifted to the right as far as we want. It is enough to require

(7) $$P_{\pi }^{t}=P_{\pi }^{t+1}\;\;\mathrm{for}\;\mathrm{all}\;\mathrm{finite}\;\mathrm{patterns}\;\pi \;\mathrm{and}\;t=1,2,\ldots$$

It is even enough to require $P_{\eta }^{1}=P_{\eta }^{2}$ for all patterns η of any length. (To prove that this implies (7) for a fixed *t* and a pattern π of length k, consider all patterns η of length m=t+k−1, which show the pattern π on their last *k* positions. The sum of their probabilities $P_{\eta }^{1}$ equals $P_{\pi }^{t}$, since *P* is a measure. And shifting all η from 1 to 2 means shifting π from *t* to t+1.)

On the other hand, infinite patterns require a limit T→∞. Moreover, there are many recent papers on infinite permutations, that is, one-to-one mappings of N onto itself. An overview was given by Pitman and Tang [26]. However, an order on N is a much wider concept than a permutation on N. An infinite permutation $\pi_{1},\pi_{2},\ldots$ defines an order on N, with the special property that below a given value $\pi_{k}$, there are only finitely many other values, for any k.

For an order on N, however, the smallest object rarely exists. Usually, each object has infinitely many other objects below and above. Nevertheless, an order on N is uniquely defined by patterns $\pi^{m}\in S_{m}$, which represent the order of the first *m* elements, for m=2,3,… for example, 12−231−3412−45132−…

**Theorem** **2** (Approximating random order on N).

*(i)*  *A sequence of permutations $\pi^{m}\in S_{m}$ defines an order on N if for m=2,3,…, the pattern $\pi^{m}$ is represented by the first m values of the pattern $\pi^{m+1}.$*
*(ii)*  *A sequence of probability measures $P^{m}$ on $S_{m}$ defines a random order P on N if for m=2,3,…, for $\pi \in S_{m}$ and $\pi^{m+1}\in S_{m+1}$, the following holds:*
  $$P_{m+1}\left\{\pi^{m+1}\;shows\;pattern\;\pi \;at\;its\;first\;m\;positions\right\}=P_{m}\left(\pi \right)\;.$$
*(iii)*  *The random order P defined by the $P_{m}$ is stationary if and only if, for m=2,3,…, for $\pi \in S_{m}$, and $\pi^{m+1}\in S_{m+1}$, the following holds:*
  $$P_{m+1}\left\{\pi^{m+1}\;shows\;pattern\;\pi \;at\;its\;last\;m\;positions\right\}=P_{m}\left(\pi \right)\;.$$

**Proof.**  (i): The condition says that the pattern of the first *m* objects, defined in step m, will not change during successive steps. So the construction is straightforward. The rank numbers, however, may change in each step, and they may converge to *∞* for m→∞.

(ii): The condition says that the probability for a pattern $\pi \in S_{m}$ to appear for the first *m* objects, defined by $P_{m},$ will remain the same for $P_{m+1}$ and successive probability measures. So we can define

$$P\left\{\mathrm{the}\;\mathrm{objects}\;\mathrm{with}\;\mathrm{numbers}\;1,\ldots ,m\;\mathrm{show}\;\mathrm{pattern}\;\pi \right\}=P_{m}\left(\pi \right)$$

in a consistent way, and $P_{m}$ determines P. Moreover, *P* is an inverse limit of the measures $P_{m}.$ Below, we provide a more elementary argument.

(iii): Together with (ii), this condition says that $P_{\pi }^{1}=P_{\pi }^{2}$ for all patterns π of any length m. As noted above, this is just the definition (7) of order stationarity. □

### 3.4. The Space of Random Orders

Resuming the discussion in Section 1.5, we show that the set of all orders on N can be represented by the unit interval, using a numeration system similar to our decimal numbers. We first assign subintervals of I=[0,1] to the permutations of length 2,3,… the pattern 12 corresponds to the interval $[0,\frac{1}{2}],$ and 21 to $[\frac{1}{2},1].$ The permutations 123, 132, and 231, which exhibit the pattern 12 at their first two positions, correspond to $\left[0,\frac{1}{6}\right],\left[\frac{1}{6},\frac{1}{3}\right],$ and $[\frac{1}{3},\frac{1}{2}],$ respectively. For intervals of length 4, see Figure 3.

Instead of the lexicographic order, we define a hierarchical order of permutations, with respect to the patterns shown by the first 2,3,…elements. For any permutation $\pi =\pi_{1}\ldots \pi_{m}$ of length *m* and any *k* with 2≤k≤m, let $r_{k}\left(\pi \right)$ denote the number of j<k with $\pi_{j}>\pi_{k}.$ This is a kind of rank number of $\pi_{k}$ with values between 0 and k−1. For example, $r_{3}\left(123\right)=0,r_{3}\left(132\right)=1,$ and $r_{3}\left(231\right)=2$ while $r_{2}=0$ for these three patterns. Now, we assign to the permutation π of length *m* the following interval:

(8) $$I\left(\pi \right)=\left[x\left(\pi \right),x\left(\pi \right)+\frac{1}{m!}\right]\;\mathrm{with}\;x\left(\pi \right)=\sum_{k=2}^{m}\frac{r_{k}}{k!}\;.$$

It is easy to check that the patterns $\pi^{\left(k\right)}=\pi_{1}\ldots \pi_{k}$ of the first k<m items of π are assigned to larger intervals, i.e.,

$$I⊃I\left(\pi^{\left(2\right)}\right)⊃I\left(\pi^{\left(3\right)}\right)⊃\ldots ⊃I\left(\pi^{\left(m\right)}\right)$$

where $\pi^{\left(m\right)}=\pi .$ Moreover, π need not be a permutation, just a pattern—it could also be a numerical time series. Only the ordering of $\pi_{j}$ is used for defining $r_{k}$ and I(π).

When we extend the pattern to the right, we obtain smaller nested subintervals, and for m→∞ a single point $x=\sum_{k=2}^{\infty }\frac{r_{k}}{k!}$, which characterizes the limiting order of infinitely many objects. Thus, each order on N corresponds to a unique point *x* in [0,1]. This is very similar to decimal expansions, where we subdivide an interval into 10 subintervals. In the case of patterns, values $r_{k}$ are the digits, and we subdivide first into 2, then into 3, 4, 5,…intervals. The endpoints of intervals represent two orders on N, but this is an exception, as 0.5 and 0.4999… for the decimals.

Once we represent all orders on N as points in [0,1], we can better understand the probability measures $P_{2},P_{3},\ldots$ of Theorem 2 and the limiting probability measure *P*, which is called the random order on N. We start with the function $F_{1}\left(x\right)=1$, which denotes uniform distribution on [0,1]. The function $F_{m}$ represents the measure $P_{m},$ for m=2,3,… for patterns π of length *m*, it is defined as the histogram of $P_{m}:$

(9) $$F_{m}\left(x\right)=m!\cdot P_{m}\left(\pi \right)\;\;\mathrm{for}\;x\in I\left(\pi \right)\;.$$

See Figure 3. The rectangle over I(π) has area $P_{m}\left(\pi \right).$ In the case of white noise, $F_{m}=1$ for all m, and $\lim F_{m}=F=1$ is the uniform distribution. We now show that such a limit exists for all sequences $F_{2},F_{3},\ldots$ for which $P_{2},P_{3},\ldots$ fulfill condition (ii) of Theorem 2. We reformulate (ii) as

(10) $$\int_{I(\pi )}F_{m+1}\left(x\right)dx=m!\cdot P_{m}\left(\pi \right)=\int_{I(\pi )}F_{m}\left(x\right)dx\;.$$

The second equation is obvious, and the first is best illustrated by an example with m=3 and π=312. We have $r_{2}=r_{3}=1,$ so $I\left(\pi \right)=\left[\frac{4}{6},\frac{5}{6}\right].$ The possible extensions $\pi^{4}$ of π are 3124, 4123, 4132, and 4231. Their intervals of length $\frac{1}{24}$ partition I(π). Condition (ii) states that $P_{4}\left\{3124,4123,4132,4231\right\}=P_{3}\left\{312\right\}.$ Thus, the four rectangles of $F_{4}$ over I(π) together have the same area as the one rectangle of $F_{3}.$ This is expressed in (10).

Equation (10) states that the $F_{m}$ form a martingale. The martingale convergence theorem implies that there is a limit function F, in the sense that $\int_{0}^{1}\left|F_{m}-F\right|dx$ converges to zero for m→∞. This limit function is integrable and ∫F=1. As a density function, it defines the probability measure *P* on all orders on N.

Our argument indicates that random orders on N belong to the realm of classical analysis and probability. Of course, the density function *F* is terribly discontinuous and can hardly be used to discuss stationarity. (An open challenge for experts is to identify examples for which the sequence $F_{m}$ converges in $L_{2}.$)

### 3.5. Extension of Pattern Distributions

We conclude our paper with an optimistic outlook for practitioners. When you find a distribution of pattern probabilities of length 3 or 4, say, from data, say, there is no need to worry about extending it to longer finite or infinite patterns. Such an extension will always exist.

**Theorem** **3** (Markov extension of pattern probabilities). *Any stationary probability measure $P_{m}$ on $S_{m}$ can be extended to a stationary probability measure $P_{m+1}$ on $S_{m+1}$, and, hence, also to a stationary probability measure P on the space of random orders.*

**Proof.**  To show that $P_{m+1}$ is stationary, we only need to verify condition (iii) of Theorem 2; any pattern within {1,…,m} can be shifted to the right, without changing its probability, until its maximum reaches m, by the stationarity assumption of $P_{m}.$ What remains is to shift the maximum from *m* to m+1, and this is condition (iii).

In the following extension formula, we use the convention that probability measures on permutations also apply to patterns, by replacing a pattern with its representing permutation. Let $\pi =\pi_{1}\pi_{2}\ldots \pi_{m}\pi_{m+1}$ be a permutation in $S_{m+1}.$

(11) $$P_{m+1}\left(\pi \right)=\frac{P_{m}\left(\pi_{1}\ldots \pi_{m}\right)\cdot P_{m}\left(\pi_{2}\ldots \pi_{m+1}\right)}{P_{m}\left(\pi_{2}\ldots \pi_{m}\right)}$$

This formula is used whenever there exists some $\pi_{k}$ with 2≤k≤m between $\pi_{1}$ and $\pi_{m+1}.$ However, if $\pi_{1}$ and $\pi_{m+1}$ are neighboring numbers, then the right-hand side of (11) cannot distinguish π and $\pi^{\prime }=\pi_{m+1}\pi_{2}\ldots \pi_{m}\pi_{1}.$ In such cases, both π and $\pi^{\prime }$ are assigned half of the value of the right-hand side, in order to avoid double counting.

The denominator on the right refers to a pattern $\pi_{2}\ldots \pi_{m}$ that has a representing permutation $\kappa =\kappa_{1}\ldots \kappa_{m-1}$ in $S_{m-1}$ and can be extended in *m* ways to a permutation $\eta =\eta_{1}\ldots \eta_{m}\in S_{m}.$ Indeed, $\eta_{m}$ can be chosen from {1,…,m}. For j<m, either $\kappa_{j}<\eta_{m}$ and $\eta_{j}=\kappa_{j},$ or $\kappa_{j}\geq \eta_{m}$ and $\eta_{j}=\kappa_{j}+1.$ Let us write

$$P_{m}\left(\kappa \right)=P_{m}\left\{\eta \;|\;\eta_{1}\ldots \eta_{m-1}\;\mathrm{shows}\;\mathrm{pattern}\;\kappa \right\}\sum_{\eta }P_{m}\left(\eta \right)\;.$$

In the numerator, $\pi_{1}\ldots \pi_{m}$ is also a pattern that has a representing permutation $\lambda =\lambda_{1}\ldots \lambda_{m}$ in $S_{m}.$ This term is $P_{m}\left(\lambda \right).$

We now prove that the defined $P_{m+1}$ fulfills condition (ii) of Theorem 2. We calculate

$$p=P_{m+1}\left\{\pi_{1}\ldots \pi_{m+1}\;|\;\pi_{1}\ldots \pi_{m}\;\mathrm{is}\;\mathrm{represented}\;\mathrm{by}\;\lambda \right\}$$

using the definition (11) of $P_{m+1}.$ There are m+1 permutations $\pi \in S_{m+1}$ that fulfill the condition, differing in the value $\pi_{m+1}.$ For each case, $\pi_{2}\ldots \pi_{m+1}$ is represented by one of the permutations η introduced above. However, the two cases π with $|\pi_{1}-\pi_{m+1}|=1$ belong to the same η. This is why we consider them together and divide their probability by two. Now,

$$p=\frac{P_{m}\left(\lambda \right)}{P_{m}\left(\kappa \right)}\cdot \sum_{\eta }P_{m}\left(\eta \right)=P_{m}\left(\lambda \right)\;.$$

This proves that $P_{m+1}$ extends $P_{m}.$ For the stationarity, the same proof has to be performed with the extension to the left and condition (iii). We must be careful that $P_{m}\left(\kappa \right)$ now refers to $\eta_{2}\ldots \eta_{m}.$ However, since we assume that $P_{m}$ is stationary, this is the same number, and the proof runs as above. □

We call this a Markov extension since (11) states that $\pi_{m+1}$ does not depend on $\pi_{1}.$ Since we do not assume any independence properties of $P_{m},$ we cannot expect more. However, if we start with $P_{2}\left(12\right)=P_{2}\left(21\right)=\frac{1}{2}$ and extend successively to m=3,4,… we obtain the coin-tossing order.

There are many other extensions. To provide an example, we can divide the double cases in an asymmetric way. A careful study of extensions may lead to a better model than coin tossing. But we have to stop here.

## 4. Conclusions and Outlook

Established models of stochastic processes do not say much about the probabilities of ordinal patterns. It has been suggested that models for ordinal pattern analysis can be found by algorithms for comparison and ranking of objects rather than by arithmetical operations. A paradigmatic example of an ordinal process without numerical values shows that this is possible. Properties like stationarity and self-similarity can be formulated in a weak and very natural way for ordinal processes. As a starting point for further work, we have proved a representation theorem and an extension theorem for stationary orders.

Our example can be improved and generalized in different ways. One way is to define ordinal processes directly from the extension theorem. Another way is to modify the algorithm so that order depends not only on coin tossing but also on already existing comparisons. Furthermore, we can consider the $P_{\pi }\left(d\right)$ of the coin-tossing order as d→∞ and prove the conjecture that this leads to an order self-similar process, similar to how the simple random walk leads to Brownian motion. On the practical side, we have to study large datasets and estimate the $P_{\pi }\left(d\right)$ for m=4 and various values of *d* as accurately as possible, in order to classify different behaviors. The fundamental mathematical challenge is to understand connections between pattern probabilities for different scales, d.

## Data Availability

Data are contained within the article. Further inquiries can be directed to the author.
